# Lincp21-RNA as Predictive Response Marker for Preoperative Chemoradiotherapy in Rectal Cancer

**DOI:** 10.3390/jpm11050420

**Published:** 2021-05-16

**Authors:** Jose Carlos Benitez, Marc Campayo, Tania Díaz, Carme Ferrer, Melissa Acosta-Plasencia, Mariano Monzo, Luis Cirera, Benjamin Besse, Alfons Navarro

**Affiliations:** 1 Department of Cancer Medicine, Gustave Roussy Cancer Center, 94805 Villejuif, France; josecarlos.benitez-montanez@gustaveroussy.fr (J.C.B.); benjamin.besse@gustaveroussy.fr (B.B.); 2 Department of Medical Oncology, Mutua Terrassa University Hospital, University of Barcelona, 08221 Terrassa, Spain; lcirera@mutuaterrassa.es; 3 Molecular Oncology and Embryology Laboratory, Human Anatomy Unit, Faculty of Medicine and Health Sciences, University of Barcelona, IDIBAPS, 08036 Barcelona, Spain; tdiaz@ub.edu (T.D.); melissaacostaplasencia@gmail.com (M.A.-P.); mmonzo@ub.edu (M.M.); 4 Department of Pathology, Mutua Terrassa University Hospital, University of Barcelona, 08221 Terrassa, Barcelona, Spain; carmeferrer@mutuaterrassa.es; 5 Faculty of Science, Orsay Campus, Paris-Saclay University, 91400 Orsay, France

**Keywords:** lincRNA-p21, rectal cancer, chemoradiotherapy, colorectal cancer, long non-coding RNA, p53, predictive biomarker

## Abstract

Preoperative chemoradiotherapy (CRT) is a standard treatment for locally advanced rectal cancer (RC) patients, but its use in non-responders can be associated with increased toxicities and resection delay. LincRNA-p21 is a long non-coding RNA involved in the p53 pathway and angiogenesis regulation. We aimed to study whether lincRNA-p21 expression levels can act as a predictive biomarker for neoadjuvant CRT response. We analyzed RNAs from pretreatment biopsies from 70 RC patients treated with preoperative CRT. Pathological response was classified according to the tumor regression grade (TRG) Dworak classification. LincRNA-p21 expression was determined by RTqPCR. The results showed that lincRNA-p21 was upregulated in stage III tumors (*p* = 0.007) and in tumors with the worst response regarding TRG (*p* = 0.027) and downstaging (*p* = 0.016). ROC curve analysis showed that lincRNA-p21 expression had the capacity to distinguish a complete response from others (AUC:0.696; *p* = 0.014). LincRNA-p21 was shown as an independent marker of preoperative CRT response (*p* = 0.047) and for time to relapse (TTR) (*p* = 0.048). In conclusion, lincRNA-p21 is a marker of advanced disease, worse response to neoadjuvant CRT, and shorter TTR in locally advanced RC patients. The study of lincRNA-p21 may be of value in the individualization of pre-operative CRT in RC.

## 1. Introduction

Rectal cancer (RC) accounts for approximately one-third of all colorectal tumors (CRC) and remains the third most common cancer worldwide and the second leading cause of cancer-related death in the world [1]. RC differs in etiologies and risk factors due to odd environmental exposures [2,3] and may have unique genetics and epigenetics factors [4]. However, during the past decade, reduction in mortality for RC has slowed [1] owing to a high rate of distant metastasis (29–39%) [5,6]. Long-term analysis has shown that preoperative chemoradiotherapy (CRT) followed by surgery of primary tumor results in persistent local control [5] and has become the standard of care for locally advanced tumors (T3-T4 or N+) [7]. The most frequently used chemotherapy agent is 5-fluorouracil in combination with concurrent fractionation radiotherapy [7]. Preoperative CRT achieves a higher radiosensitivity of tissues before surgery, a lower rate of toxicities, and a higher probability of sphincter preservation due to tumor downstaging [8]. Of note, the rate of pathological response after neoadjuvant treatment has been associated with prognosis [8,9]. Pathological complete response (pCR; ypT0N0), which occurs in 15–25% of patients, has been linked with lower rates of local recurrences [9,10]. Indeed, to achieve a complete response after preoperative CRT has been associated with better disease-free survival (DFS) and overall survival (OS) rates [9]. Nonetheless, survival outcomes of patients with an assessed pCR compared to those without have not been properly compared; therefore, selection of patients to avoid unnecessary toxicities and to perform suitable management remains uncertain. Furthermore, despite the adoption of adjuvant postoperative chemotherapy, patients are more than twice as likely to present with a distant recurrence rather than tumor regrowth at the primary site [5,6]. This situation emphasizes the urgency of devising upfront treatment strategies aimed at controlling obscure micro-metastases. Identifying patients who will not respond to treatment is crucial to avoid unnecessary treatment, potential toxicities, and a delay of surgery. Biomarkers to identify patients at high risk of relapse or lack of response are needed to guide treatment options and improve survival rates [11], and non-coding RNAs are promising candidates [12,13].

Non-coding RNAs comprise 97% of the transcriptome, while protein-coding messenger RNAs (mRNA) account for only 3% [14]. Long non-coding RNAs (lncRNAs) have been related to the main hallmarks of cancer [15] and have been described as key in the tumorigenesis of different solid tumors, including RC [16,17]. Indeed, lncRNAs have been shown to be highly tissue specific [18,19], being able to discriminate between tumor and normal cells [20]. The long intergenic non-coding RNA p21 (lincRNA-p21) acts as a regulator for p53-mediated apoptosis [21], angiogenesis [22], and HIF1A-mediated response to hypoxia in cancer cells [23]. However, the role of lincRNA-p21 in RC remains poorly understood and explored only in vitro or using small cohorts of patients [24,25]. In this setting, lncRNAs, and especially lincRNA-p21, could serve as predictive biomarkers to select the most optimal treatment in each case in order to individualize therapy. We aimed to evaluate whether lincRNA-p21 can act as a predictive biomarker for CRT response in a 70-patient cohort of RC treated before resection.

## 2. Materials and Methods

### 2.1. Study Population

Seventy patients diagnosed from December 2006 to October 2016, with RC and available baseline endoscopy biopsy from Mutua Terrassa University Hospital, were included in the present study. All selected patients suffered with rectal adenocarcinoma in a clinical stage II or III (uT3-T4 and/or uN+) and were consecutively treated at Mutua Terrassa University Hospital. Although the study population was collected in Barcelona (Europe), ethnical information was not considered for patient inclusion within the study. All samples were stored as paraffin-embedded blocks until use. All patients had received neoadjuvant chemotherapy with 5-fluorouracil 225 mg/m^2^/day × 7 days in continuous infusion and in combination with pelvic locoregional radiotherapy (45–50 Gy). Six to eight weeks after completion, all patients underwent surgery. All surgical specimens were evaluated and classified according to TNM 7th edition, and the pathological response was graded according to the tumor regression grade (TRG) Dworak classification [26]. Approval for the study was obtained from the Institutional Review Board of the Mutua Terrassa University Hospital, Barcelona, Spain.

### 2.2. RNA Extraction and lincRNA-p21 Quantification

Total RNA was extracted from formalin-fixed, paraffin-embedded, tumor tissues from pretreatment endoscopy biopsies using a RecoverAll Total Nucleic Acid Isolation Kit (Ambion, ThermoFisher Scientific, Waltham, MA, USA) as previously reported [27] and quantified using a NanoDrop ND-1000 Spectrophotometer (NanoDrop Technologies, Wilmington, DE). Total cDNA was obtained from 250 ng of RNA using the High-Capacity cDNA Reverse Transcription Kit (Applied Biosystems, Foster City, CA, USA). LincRNA-p21 expression was determined as previously described [22]. LincRNA-p21 expression was calculated using 2^−ΔΔCt^ using B2M (beta-2-microglobulin) (Hs99999907_m1) (Applied Biosystems) as endogenous control.

### 2.3. Statistical Methods

Assumptions of distributional normality were tested using the Shapiro–Wilk test and quantile–quantile plot. Continuous data were tested with the T-test (two groups) or ANOVA (more than two groups) when normally distributed and the Mann–Whitney U-test or Kruskal–Wallis test when not normally distributed. ROC curves were calculated using R package pROC [28]. The multivariate analysis for treatment response was performed by using binary logistic regression. Time to relapse (TTR) was defined as the time between resection and recurrence or last follow-up. Overall survival (OS) was calculated from the time of resection to the date of death or last follow-up. Optimal cutoffs of lincRNA-p21 expression data for TTR and OS were obtained using X-Tile software [29]. Kaplan–Meier curves for TTR and OS were plotted and compared with log-rank test. The multivariate analysis was performed using the stepwise proportional hazard Cox regression model to determine hazard ratios (HR) with their 95% confidence intervals (CI). Statistical significance was set at *p* ≤ 0.05. All statistical analyses were performed using IBM SPSS Statistics 26 (SPSS Inc., IBM, Chicago, IL, USA), R 4.0.2 and GraphPad Prism v9.1.0.

## 3. Results

### 3.1. Patient Characteristics

Samples from 70 patients were analyzed, most of whom were males (*n* = 49, 70%). Median age at diagnosis was 66 (range: 38 to 82) years. Sixty-one (87.1%) patients reported stage III and 9 (12.9%) stage II; 52 (74.3%) patients were assessed for TRG 0–3, and 18 (25.7%) reported pathological complete response (TRG 4, ypT0N0); 64.3% of downstaging was shown. Finally, 43 (66.2%) patients received adjuvant chemotherapy after primary tumor resection. Table 1 shows further main characteristics of the 70 patients included in the study. Median follow-up time was 105.40 months (IQR: 78.63–127.33).

### 3.2. LincRNA-p21 Expression Levels

**Figure 1:**
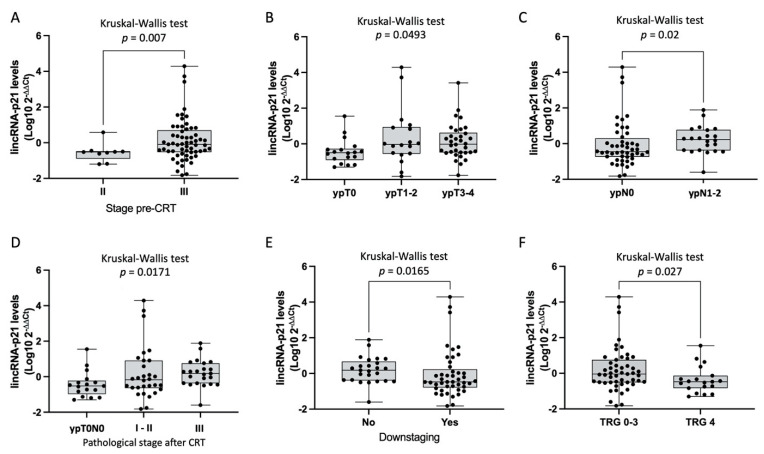
LincRNA-p21 levels and clinicopathological characteristics. (**A**) LincRNA-p21 expression in (**A**) stage III vs. stage II; (**B**) ypT0 vs. ypT-1–2 vs. ypT3–4; (**C**) ypN0 vs. ypN1–2; (**D**) ypT0N0 vs. I-II vs. III; (**E**) downstaging no vs. yes; (**F**) TRG 0–3 vs. TRG 4.

The correlation of lincRNA-p21 levels in tumor tissue with the main clinicopathological characteristics showed a significant association with disease stage, ypT, ypN, pathological stage (ypTNM), downstaging, and pathological response. LincRNA-p21 was upregulated in stage III compared to stage II tumors (*p* = 0.007) (Figure 1A). Significant differences in lincRNA-p21 levels were observed according to ypT, where the ypT0 group had the lowest levels (*p* = 0.0493, Figure 1B). Patients with ypN1–2 showed higher levels of lincRNA-p21 (*p* = 0.02). Furthermore, patients with pathological stage III had higher lincRNA-p21 levels (*p* = 0.0171). Tumors with the worst response to CRT regarding negative downstaging and TRG 0–3 showed higher levels of lincRNA-p21 than tumors with positive downstaging (*p* = 0.0165; Figure 1E) and TRG4 (TRG0–3, *n* = 52 vs. TRG4, *n* = 18, *p* = 0.027; Figure 1F).

### 3.3. Predictive Ability of lincRNA-p21 for Response to CRT

**Figure 2:**
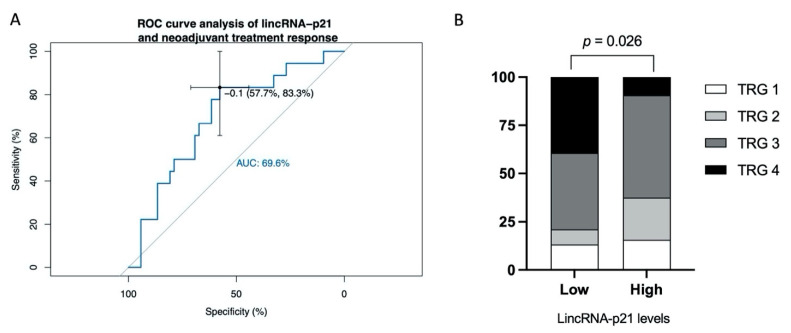
Predictive analyses for response to neoadjuvant treatment. (**A**) ROC curve analyses to evaluate the potential utility of lincRNA-p21 to distinguish patients with maximum response to neoadjuvant treatment (TRG4) from others (TRG 0–3). (**B**) Percentage of patients with each TRG according to low vs. high lincRNA-p21 expression, dichotomized using optimum truncation point obtained in the ROC curve analysis (−0.1). AUC, area under the curve. TRG, tumor regression grade.

Receiver operating characteristic (ROC) curves were generated to investigate the potential of lincRNA-p21 as a marker for neoadjuvant treatment response. The area under the curve (AUC) value showed that lincRNA-p21 expression had capacity to distinguish patients with complete response (TRG4) from others (AUC: 0.696; 95% confidence interval (CI) = 0.558–0.833; *p* = 0.014). In the optimum truncation point (−0.1), the sensitivity and specificity were 83.3% and 57.7%, respectively (Figure 2A). Using the best threshold identified by the ROC curve analysis, we divided the patients into two groups, observing that there were differences in TRG proportions allocation between low or high lincRNA-p21 levels (*p* = 0.026, Figure 2B). Among patients with low levels of lincRNA-p21, 39.5% had a TRG 4 vs. only 9.4% in the group with a high lincRNA-p21 expression value.

Finally, we performed a multivariate analysis of response to neoadjuvant treatment including sex, age, pre-CRT stage, CEA levels pre-CRT, and lincRNA-p21 levels (Table 2). Only lincRNA-p21 levels emerged as an independent marker of neoadjuvant treatment response (odds ratio (OR): 0.485; 95% CI: 0.237–0.992; *p* = 0.047).

### 3.4. LincRNA-p21 Expression and Survival

In our cohort, overall, median TTR and median OS were not reached (NR). Overall, mean TTR was 136.5 months (95% CI: 127.8–145.2) and mean OS was 124.3 months (95% CI: 114–134.6).

**Figure 3:**
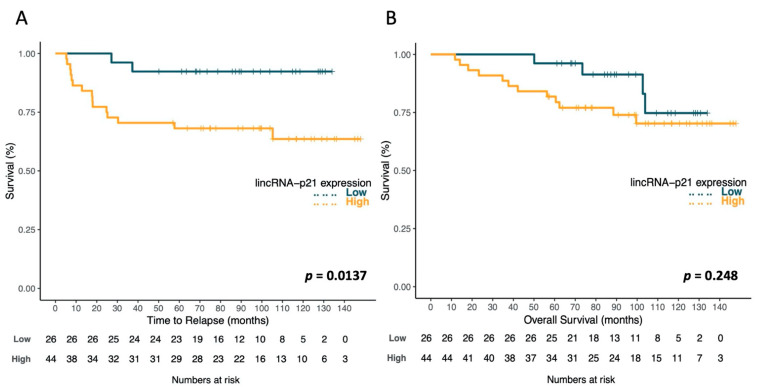
Kaplan–Meier curves for time to relapse (TTR) (**A**) and overall survival (OS) (**B**) according to lincRNA-p21 expression levels in 70 rectal cancer patients. The log-rank test was used to calculate whether significant differences in survival times between high or low lincRNA-p21 levels were achieved.

Using the optimal cutoff values identified by X-Tile, the patients were classified in two groups as having high or low lincRNA-p21 levels. Among the 70 RC patients, 26 were classified as low, and 44 as high. Patients with high lincRNA-p21 levels had significantly shorter TTR (*p* = 0.014). TTR for patients with high levels was 104.4 months (95% CI 86.4–122.5), while it was 126.2 months (95% CI 115.7–136.6) for those with low levels (Figure 3A). No significant differences were observed for OS (*p* = 0.284), but patients with high lincRNA-p21 levels had shorter OS (116.9 vs. 129.5 months; Figure 3B).

### 3.5. Multivariate Analysis of TTR and OS

In the univariate analysis, there were statistically significant differences in TTR and OS related to tumor pathological stage (ypT), lymph node pathological stage after CRT (ypN), pathological stage after CRT (ypTNM), and downstaging. The *p*-values are summarized in Table 1. Since ypT and ypN are included in the calculation of pathological stage, ypTNM, we decided to include only the pathological stage, downstaging, and the lincRNA-p21 expression in the Cox multivariate analysis (Table 3). The multivariate analysis showed that lincRNA-p21 levels (HR, 4.458;95% CI, 1.014–19.603; *p* = 0.048) and stage (HR, 4.430; 95% CI: 1.266–15.497; *p* = 0.020) were independent prognostic factors for TTR, while downstaging (HR, 3.512; 95% CI: 1.275–9.673; *p* = 0.015) was the unique independent prognostic factor for OS.

## 4. Discussion

We showed the potential use of lincRNA-p21 expression levels in tumor tissue from baseline biopsies of RC patients as a predictive marker of CRT response and as a prognostic biomarker for TTR. Firstly, we observed that higher lincRNA-p21 levels were found in patients with stage III pre-CRT, and, interestingly, after CRT treatment, the highest lincRNA-p21 levels were reported for patients presenting pathological stage III, and the lowest levels were found in patients with ypT0N0. Indeed, higher lincRNA-p21 levels were observed in patients with ypT3–4 and in ypN1–2 patients. These results are in line with previous reports in CRC [17,25]. In a cohort of 66 patients with CRC, including 39% (26/66) of RC [25], higher lincRNA-p21 levels were associated with poor prognostic factors, such as a poorer stage (stage III vs. I), tumor size (pT), and vascular invasion [25]. In another study, Li et al. analyzed 177 CRC tumors samples from surgical resection, of which 81 (45.7%) were RC; lincRNA-p21 was found as a marker of advanced disease, as higher lincRNA-p21 levels were observed in stage III patients and in N+ patients, and worse survival [17]. However, although these reports are in line with our results, we must take into account that we studied a different RC population, namely, patients receiving neoadjuvant treatment before surgery. Of note, this group of patients was excluded from both previous reports.

Secondly, we observed that lower lincRNA-p21 levels were found in patients who underwent tumor downstaging and complete pathological response after CRT treatment. Locally advanced rectal cancer patients are commonly explored with a rectal endoscopy, which provides sufficient tissue samples for diagnosis and biomarker analyses. Currently, there are no clinically validated biomarkers to correctly identify those patients that will not respond. LincRNA-p21 emerged as a predictive biomarker for CRT response, and when it was compared to other predictive factors at diagnosis such as baseline stage or CEA levels, it was shown as an independent predictor factor. The neoadjuvant CRT treatment in our cohort was based on 5-fluorouracil combined with locoregional radiotherapy. Wang et al. carried out an in vitro study aiming to evaluate the role of lincRNA-p21 in radiotherapy response [24]; in contrast to our results, they described that lincRNA-p21 expression level may affect the sensitivity to radiotherapy. In this study, the authors observed that after X-ray treatment, the levels of lincRNA-p21 became upregulated in two colorectal cancer cell lines, SW1116 and LOVO. When researchers overexpressed lincRNA-p21 in the SW1116 cell line and treated the cells with X-rays, they noted a higher apoptosis rate than in control cells; nonetheless, this result was not validated by the authors when they silenced lincRNA-p21 before X-ray treatment on the same cell line (no differences in apoptosis rate were observed between the silenced and control group). Our group has reported results in this line; however, we used a different cohort of patients (resected CRC patients not receiving neoadjuvant treatment) [17]. We observed that patients with tumors with high expression of lincRNA-p21 demonstrated an increased benefit of CRT as an adjuvant therapy (longer OS compared to those patients not receiving CRT after surgical resection [17]). Nonetheless, these results are not comparable with the present work since our correlation was obtained in tumor tissue isolated prior to adjuvant CRT treatment administration. Moreover, the two previous references focused on the potential role of lincRNA-p21 and radiotherapy response in relation to its role in the p53 pathway [21,30,31,32]; nonetheless, we cannot ignore that rectal cancer patients included in the cohort also received 5-fluorouracil. Lee and colleagues analyzed the pattern of lncRNAs in 5-fluorouracil-resistant colon cancer cell lines and observed that lincRNA-p21 was significantly upregulated in SNU-C5 5-FU-resistant cells compared to its parental cell line [33]. This provides an important insight into the involvement of lincRNA-p21 within 5-FU resistance of colon cancer cells and allows us to speculate the following: the better response rates observed in patients with low levels of lincRNA-p21 could be associated with, at least partially, an enhanced sensitivity to 5-fluorouracil. The role of lincRNA-p21 in 5-fluorouracil resistance and its effect when 5-FU is combined with radiotherapy deserves further study, but this is out of the scope of the present paper.

Finally, we found a correlation between high expression of lincRNA-p21 levels and shorter TTR. In this regard, high lincRNA-p21 levels have been previously related to a worst outcome in CRC [17] and also in other solid tumors such as non-small-cell lung cancer [22], bladder carcinoma [34], or hepatocellular carcinoma [35]. In CRC, Li et al. observed that lincRNA-p21 was found as a marker of advanced disease and worse survival outcomes, especially for RC where high lincRNA-p21 levels were linked to shorter DFS and shorter OS [17].

We are conscious that the present study has several limitations, including the small number of samples analyzed (*n* = 70), which can affect the robustness of the multivariate analysis. The results obtained in the multivariate analysis, despite being informative, need to be validated in a larger cohort. Moreover, an additional limitation is that lincRNA-p21 was analyzed in a retrospective cohort of paraffin-embedded samples. Nonetheless, no related studies have been published for RC patient cohorts in neoadjuvant settings, and our study may provide new evidence of epigenetic pathways behind the tumor response to CRT. LincRNA-p21 may be a promising predictive biomarker of CRT benefit, avoiding delay of resection and unnecessary comorbidities for those patients with tumors and reporting high expression levels of lincRNA-p21 at baseline.

## 5. Conclusions

LincRNA-p21 is a marker of advanced disease, worse response to neoadjuvant CRT, and shorter TTR in locally advanced rectal cancer patients. The study of lincRNA-p21 in endoscopy samples obtained prior to treatment decision may be of value in the individualization of pre-operative CRT in rectal cancer.

## Figures and Tables

**Table 1:** Main clinical characteristics of the 70 patients included in the study with their associated time to relapse (TTR) and overall survival (OS) according to the univariate analyses (log rank). Significant *p*-values are shown in bold. RC: rectal cancer; CRT: Chemo-radiotherapy.

Characteristic		Number of Patients (%)	TTR *p*-Value	OS *p*-Value
Sex	Male	49 (70)	0.203	0.269
	Female	21 (30)		
Median age (range)	66 (38–82)			
	<60	19 (27.1)	0.679	0.815
	>60	51 (72.9)		
Clinical stage pre-CRT	II	9 (12.9)	0.585	0.497
	III	61 (87.1)		
Adjuvant therapy	No	27 (33.8)	0.776	0.130
	5-FLU	7 (8.7)		
	FOLFOX	40 (50)		
	Other	6 (7.5)		
ypT	ypT0	18 (25.7)	0.015	0.051
	ypT1–2	18 (25.7)		
	ypT3–4	34 (48.6)		
ypN	ypN0	48 (68.6)	0.003	0.044
	ypN1–2	22 (31.4)		
Pathological stage after neoadjuvant CRT	ypT0N0	17 (24.2)	0.024	0.133
	I	16 (22.9)		
	II	14 (20)		
	III	23 (32.9)		
Downstaging	No	25 (35.7)	0.001	0.010
	Yes	45 (64.3)		
Tumor regression grade (TRG)	0–3	52 (74.3)	0.324	0.161
	4	18 (25.7)		

**Table 2:** Results obtained in the multivariate logistic analysis for complete response to neoadjuvant treatment (TRG4 vs. others).

Factors	OR (95% CI)	*p*-Value
Stage II at diagnosis	1.703 (0.363–8.003)	0.500
Age	0.980(0.918–1.046)	0.549
Gender male	2.756 (0.682–11.137)	0.155
CEA at baseline	0.930 (0.809–1.068)	0.301
LincRNA-p21 levels	0.485 (0.237–0.992)	0.047
Constant	0.307	<0.001

**Table 3:** Multivariate analysis for TTR and OS.

**Time to Relapse**	**HR (95% CI)**	***p*** **-Value**
Pathological stage > I	4.430 (1.266–15.497)	0.020
No downstaging	1.737 (0.350–8.621)	0.499
High lincRNA-p21	4.458 (1.014–19.603)	0.048
**Overall Survival**	**HR (95% CI)**	***p*** **-Value**
Pathological stage > I	2.020 (0.362–11.273)	0.423
No downstaging	3.512 (1.275–9.673)	0.015
High lincRNA-p21	1.387 (0.411–4.679)	0.598

## Data Availability

Not applicable.
